# Grid-enhanced X-ray coded aperture microscopy with polycapillary optics

**DOI:** 10.1038/srep44944

**Published:** 2017-03-21

**Authors:** Katarzyna M. Sowa, Arndt Last, Paweł Korecki

**Affiliations:** 1 Institute of Physics, Jagiellonian University, Łojasiewicza 11, 30-348 Kraków, Poland; 2 Institute of Microstructure Technology, Karlsruhe Institute of Technology, 76344 Eggenstein-Leopoldshafen, Germany

## Abstract

Polycapillary devices focus X-rays by means of multiple reflections of X-rays in arrays of bent glass capillaries. The size of the focal spot (typically 10–100 *μ*m) limits the resolution of scanning, absorption and phase-contrast X-ray imaging using these devices. At the expense of a moderate resolution, polycapillary elements provide high intensity and are frequently used for X-ray micro-imaging with both synchrotrons and X-ray tubes. Recent studies have shown that the internal microstructure of such an optics can be used as a coded aperture that encodes high-resolution information about objects located inside the focal spot. However, further improvements to this variant of X-ray microscopy will require the challenging fabrication of tailored devices with a well-defined capillary microstructure. Here, we show that submicron coded aperture microscopy can be realized using a periodic grid that is placed at the output surface of a polycapillary optics. Grid-enhanced X-ray coded aperture microscopy with polycapillary optics does not rely on the specific microstructure of the optics but rather takes advantage only of its focusing properties. Hence, submicron X-ray imaging can be realized with standard polycapillary devices and existing set-ups for micro X-ray fluorescence spectroscopy.

Polycapillary focusing elements^1^ are frequently used in X-ray analysis for chemical mapping^2^, microimaging^3^, depth profiling^4^ and X-ray spectroscopy^5^. They have found application in many disciplines, e.g., biology^6^, materials science^7^, earth science^8^, investigations of cultural heritage^9^ and forensic science^10^. A polycapillary optics consists of up to one million hollow, curved and tapered glass capillaries, in which X-rays are transmitted by means of multiple total external reflections. The focal spot of the optics is formed by the incoherent overlapping of the beams from the individual capillaries. The size of the focal spot is determined by the working distance of the optics or its focal length *f* and by the divergence of the microbeams generated by the individual capillaries. The size of the focal spot can be approximated as 2*fα*_*c*_, where *α*_*c*_ is the critical angle for total external reflection. Hence, in the hard X-ray range (5–20 keV), the focal spot has dimensions ranging from 10 *μ*m to 100 *μ*m. Although this size is much larger than for other kinds of X-ray focusing devices^11^,^12^,^13^, polycapillaries have very high angular apertures, are achromatic and generate a high photon density in the focal plane. This makes them especially useful in laboratory-based experiments using X-ray tubes^14^. Nevertheless, the size of the focal spot limits the resolution not only of scanning methods but also of X-ray absorption^15^,^16^ and phase-contrast imaging^17^,^18^ using polycapillary optics, in which the focal spot acts as a secondary X-ray source.

Recent works have demonstrated that it is possible to resolve details of objects that are placed inside the focal spot of a polycapillary optics and to improve its “nominal” resolution^15^,^19^. This is made possible by means of the coded aperture principle^20^. Coded aperture imaging has its roots in X-ray and *γ*-ray astronomy^21^ and requires apertures with specially designed shapes that ensure efficient and well-posed decoding, e.g., random masks of pinholes^22^ or uniformly redundant arrays^23^. In the general case, simple periodic arrays are not suitable for this purpose. However, at the focal plane, the field of view is confined to the area of the focal spot. Hence, effectively, the size of the illuminated object is always small. The Shannnon sampling theorem^24^ predicts that for an object with a finite support, the use of a periodic coded aperture is possible unless the period of the coding pattern is larger than the effective size of the object. Therefore, in X-ray coded aperture microscopy with polycapillary optics (XCAMPO), the structure of an imaged object can be decoded from an image recorded using a capillary optics with a nearly perfectly periodic hexagonal superstructure. This hexagonal superstructure results from a specific manufacturing technology and is a hallmark of polycapillary devices^25^. In a proof-of-principle XCAMPO experiment, object details at a resolution of approx. 8 microns could be reconstructed using an optics with a 40 *μ*m spot^15^.

However, XCAMPO cannot be directly extended to submicron imaging, i.e., to imaging at a resolution comparable to the spacing of the individual capillaries, which is always much smaller than the size of the focal spot. Very recently, to overcome this problem, a special variant of XCAMPO, namely, defect-assisted microscopy^26^, was proposed. This approach takes advantage of natural point defects in polycapillary structures, such as missing, crushed or slightly larger capillaries. It was demonstrated that such intrinsic point defects, by breaking the periodicity of capillary arrays, lead directly to the formation of multiple X-ray images of an object placed inside the focal spot of a polycapillary optics. Such multiple images can be analysed using the coded aperture principle and provide a spatial resolution at the level of 0.5 *μ*m. Defect-assisted XCAMPO is very promising, but for practical applications, it requires the challenging fabrication of tailored X-ray optics with intentionally introduced defects.

In this work, we demonstrate submicron coded aperture microscopy using an external periodic grid placed at the output surface of the polycapillary optics. In contrast to previous approaches, in which the internal microstructure was used as the coded aperture, grid-enhanced X-ray coded aperture microscopy with polycapillary optics does not rely on the specific structure of a polycapillary optics. However, grid-enhanced XCAMPO still profits from the small size of the focal spot of the optics and from the high flux inside the focal spot. Therefore, submicron X-ray imaging can be realized with standard polycapillary optics or existing set-ups for micro X-ray fluorescence spectroscopy (*μ*XRF)^27^,^28^. In this work, we used a standard grid for transmission electron microscopy as the coding aperture. In future, the shape of the coded aperture could be optimized using existing micro- and nanofabrication technology.

## Results

### Image formation in grid-enhanced XCAMPO

**Figure 1:**
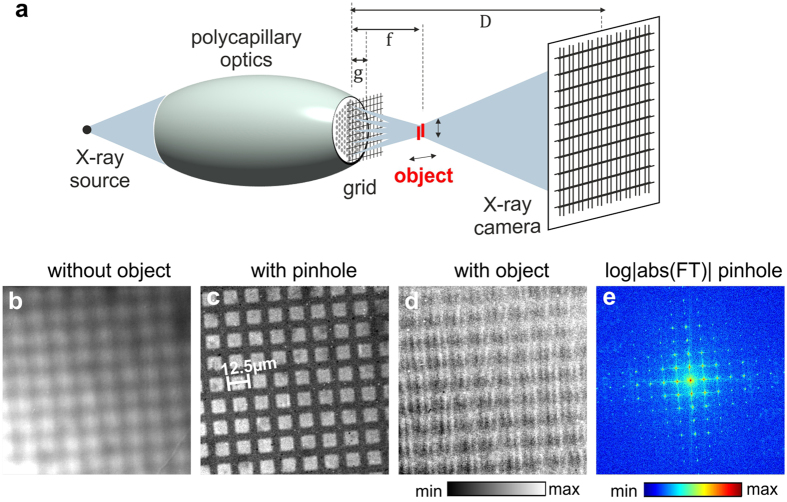
The principle of image formation in grid-enhanced X-ray coded aperture microscopy with polycapillary optics. (**a**) A sketch of the experimental geometry. A periodic grid with a pitch larger than the size of the focal spot of the optics is placed at the output surface of the optics. An X-ray camera records a magnified image of the grid. (**b**) A blurry image of the grid (mesh 2000) recorded without any object in the focal spot. (**c**) A sharp image of the grid recorded with a 1 *μ*m pinhole inside the focal spot. (**d**) A distorted image of the grid recorded with an object (vertical 2 *μ*m slits) inside the focal spot. The structure of the object can be decoded from this distorted image of the grid using image (**c**) as the coding pattern. (**e**) Fourier transform of the grid image in (**c**). Periodic peaks (up to the 7th harmonic of the grid period) are visible. The spatial resolution corresponds to the highest resolved harmonic frequency of the grid. The edges correspond to approx. *f*_*c*_/4, where *f*_*c*_ is the Nyquist frequency of the detector.

The principle of grid-enhanced XCAMPO is presented in Fig. 1(a). A periodic aperture or grid with a pitch larger than the size of the focal spot is placed at the output surface of the optics. The object to be imaged is placed inside the focal spot, and a magnified image of the grid is recorded by an X-ray camera.

For a simplified description of the image formation principle in grid-enhanced XCAMPO, a slightly modified version of a formula presented in previous works can be used^15^,^29^. For an object placed in the focal plane of the optic, the intensity *I(**r***) of the X-rays recorded by the camera can be approximated by the following formula:


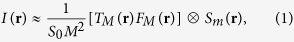


where ⊗ denotes the convolution operation; *T*_*M*_ is the transmission of the object, magnified by a factor of (1 − *M*); and the magnification factor is given by *M* = (*f* − *D*)/*f*, where *D* is the detector-to-optics distance. *F*_*M*_ describes the spatial distribution of the radiation in the focal spot (with an approximately Gaussian shape) at a magnification of *M*. Note that the polycapillary optics transmits radiation in an incoherent way. Hence, to a first approximation, the shape of the focal spot is not modified by the presence of the external grid at the output surface of the optics. *S*_*m*_ represents the spatial distribution of the radiation behind the grid at a magnification of *m*. This second magnification factor is defined as *m* = (*f* − *g* − *D*)/(*f* − *g*), where *g* is the distance between the grid and the output surface of the optics. The prefactor *S*_0_ is the total number of photons in the focal spot. Equation (1) means that an object inside the focal spot of the optics “distorts” the image of the grid recorded using the X-ray camera. In other words, the distorted image of the grid encodes information about the object.

### Proof-of-principle experiment

A proof-of-principle experiment for grid-enhanced XCAMPO was performed using a laboratory set-up. A gold transmission electron microscopy grid with a pitch of 12.5 *μ*m was placed close to the output surface (*g* ≈ 0.5 mm) of a focusing polycapillary optics with an exit diameter of 1.1 mm and a focal length *f* ≈ 2.5 mm. The FWHM of the focal spot of the optics was Δ*x* ≈ 11 *μ*m. X-rays were recorded by a scintillator that was lens coupled to an sCMOS camera. The scintillator was placed at a distance of *D* ≈ 40 cm from the optics. Figure 1(b) shows an X-ray image of the grid that was recorded without any object between the grid and the camera. The image of the grid is blurred because of the finite divergence of the microbeams exiting from the capillaries. When a pinhole was placed inside the focal spot of the optics, the image of the grid became much sharper, as demonstrated in Fig. 1(c). A pinhole approximates an object with point-like or *δ*-like transmission, for which equation (1) becomes *I* ≈ *const* × *S*_*m*_. Hence, in the image shown in Fig. 1(c), the grid is resolved at a resolution comparable to the size of the pinhole. This is evidenced in Fig. 1(e), which shows the Fourier transform of the grid image recorded with the pinhole present in the focal spot. One can observe peaks corresponding to the 7th harmonic of the grid period. Note that the sixfold symmetry corresponding to the capillary microstructure is not visible in Fig. 1(e). First, the resolution of the camera was slightly too poor to resolve individual capillaries. Second, the peaks corresponding to the superstructure of the capillary bundles were much weaker than the peaks induced by the presence of the grid. The grid image recorded in the presence of the pinhole was subsequently used in the decoding procedures as the coding pattern, *S*. When an object (set of vertical slits with widths of 2 *μ*m) was placed in the focal spot, the image of the grid become sharper and also distorted. This distorted image encodes high-resolution (not limited by the focal spot size) information about the object.

### Spatial resolution

**Figure 2:**
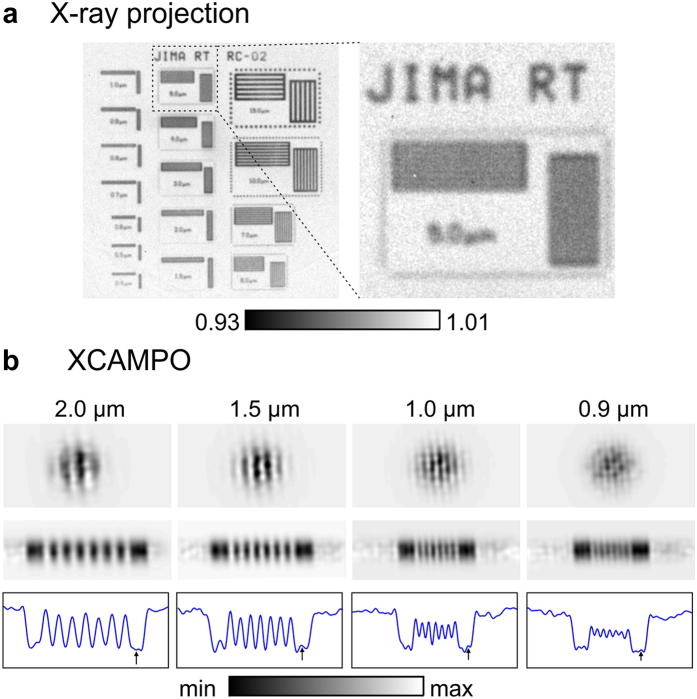
Comparison of the resolutions in conventional projection imaging and in grid-enhanced XCAMPO. (**a**) Standard X-ray projection of the JIMA resolution chart obtained without the coding grid and with the chart placed far from the focal plane of the optics. Conventional X-ray projection can resolve lines with a width of 5 *μ*m. (**b**) Decoded grid-enhanced XCAMPO images of slits of different widths in the JIMA chart. Top: decoded images from a single grid-enhanced XCAMPO exposure. Middle: one-dimensional grid-enhanced XCAMPO scans showing larger object areas. Bottom: averaged profiles of the data presented in the middle row. Grid-enhanced XCAMPO can resolve lines with a width of 0.9 *μ*m.

To test the resolution of grid-enhanced XCAMPO, we used a JIMA RT RC-02B resolution chart consisting of a set of slits of various widths in an absorbing tungsten material with a thickness of 1 *μ*m. Figure 2(a) shows an X-ray projection recorded without the coding grid and with the chart placed far from the focal plane (exposure of 600 s). The image was normalized with respect to data recorded without the chart. In the projection geometry, the focal spot of the optics acts as a secondary X-ray source, and the half-pitch resolution is limited by the size of the focal spot to approx. 5 *μ*m (i.e., to ~Δ*x*/2).

Using projection imaging, slits of various widths were moved into the focal spot of the optic to record grid-enhanced XCAMPO images (exposures of 600 s). Decoding was performed through the deconvolution of equation 1. The image recorded in the presence of the pinhole was taken as an approximation of the coding pattern *S*. A direct deconvolution of equation 1 using the coding pattern *S* yields the object’s transmission *T*, multiplied by the shape of the focal spot *F*. However, the focal spot *F* was a smooth Gaussian and its shape did not affect the details of the reconstruction. Images decoded from single grid-enhanced XCAMPO images are shown in the top row of Fig. 2(b). To improve the image quality, we performed 1D scans. Each object was scanned across the focal spot, and several grid-enhanced XCAMPO images were recorded and combined, as explained in the Methods section. The resulting data are plotted in the middle row of Fig. 2(b). The bottom row of Fig. 2(c) shows the averaged profiles of these data. These data indicate that the half-pitch spatial resolution is better than 0.9 *μ*m. The highest resolved spatial frequency (7th harmonic) of the grid image shown in Fig. 1(e) is *f*_*max*_ = 7/12.5 *μ*m^−1^. This corresponds to a half-pitch resolution of ~0.89 *μ*m. This means that the decoding procedure uses all frequencies up to *f*_*max*_ and is robust against noise. However, please note, that the spatial resolution is slightly anisotropic. For horizontal lines (data not shown), a half-pitch resolution between 1 *μ*m and 1.5 *μ*m was achieved. The observed anisotropy is most probably due to thermal drifts, which were much larger in the vertical direction than in the horizontal one, as directly observed during the system warm up phase. While a polycapillary optics is a non-imaging device, the position of the optics focal spot weakly depends on the position of the X-ray source^30^, which may change during long exposures.

### Depth resolution

**Figure 3:**
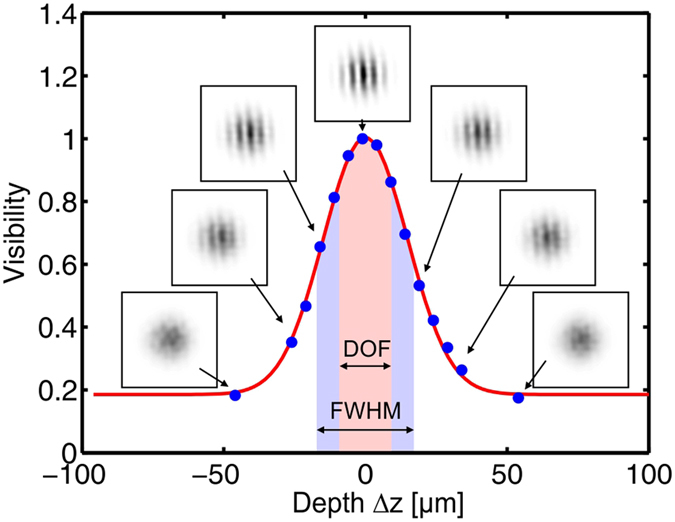
Evaluation of the depth of field in grid-enhanced XCAMPO. Each point in the graph represents the visibility of the lines decoded from grid-enhanced XCAMPO images of the 1.5 *μ*m slits in the JIMA resolution chart at various distances Δ*z* between the object and the focal plane. The insets show examples of the decoded images.

XCAMPO offers the capability of 3D layer-by-layer laminographic imaging^19^. To estimate the depth resolution of grid-enhanced XCAMPO, coded images were recorded with the object at various displacements with respect to the focal plane. For this experiment, we imaged the slits with a width of 1.5 *μ*m, for which the maximal visibility at the focal plane i.e. at Δ*z* = 0 was nearly 100% (cf. Fig. 2(b)). For finer slits, the maximal visibility was affected by the finite lateral spatial resolution. The visibility of the slits is plotted in Fig. 3 for various distances Δ*z* between the object and the focal plane. The FWHM of the fitted Gaussian is ~34.9 *μ*m, and the depth of field (which is usually evaluated at 80% of the maximum intensity^31^) is ~19.9 *μ*m. XCAMPO shares many properties with laminography^32^. For example, the depth resolution depends on the spatial frequency of the imaged features. Hence, one can estimate that for details at the finest half-pitch resolution of 0.9 *μ*m, the depth of field is at the level of 12 *μ*m. This value is much larger than the resolution in the lateral plane. This results from the finite (though very large) aperture of the optics. Note, however, that such a resolution is comparable to the resolution of confocal methods that require two “crossed” polycapillary lenses^33^.

### Image quality and decoding artefacts

**Figure 4:**
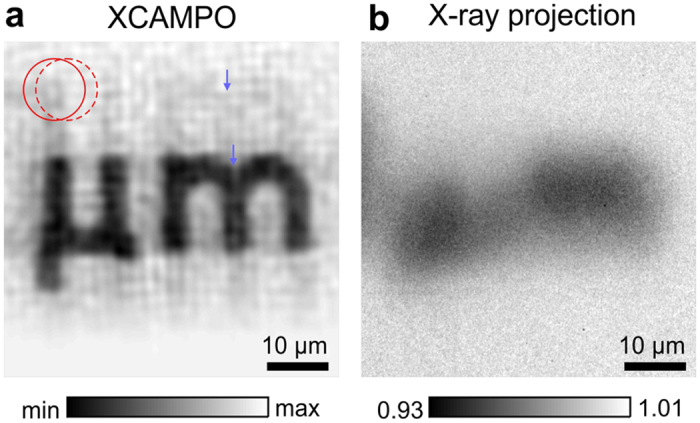
A reconstruction of a larger 2D object from a grid-enhanced XCAMPO dataset is presented in (**a**). For comparison, (**b**) shows a standard X-ray projection image obtained with the optic but without the grid. The centres of the red circles indicate two successive scanning positions, and their diameters reflect the focal spot size. The arrows indicate corresponding points in the real image and in a weak “ghost” image.

Figure 4(a) shows an image of a text label reading “*μ*m” on the JIMA chart obtained via grid-enhanced XCAMPO at a submicron resolution.

For comparison, Fig. 4(b) shows a standard X-ray projection image recorded with the optics but without the coding grid. The size of the object is much larger than the size of the focal spot. Therefore, to extend the field of view (FOV) of grid-enhanced XCAMPO, the object was laterally scanned in 2 *μ*m steps in both directions (31 × 21 scan points). In Fig. 4(a), the scan step and the FOV of a single exposure are illustrated by the position and diameter, respectively, of the red circles. The step size was intentionally chosen to be much smaller than the FOV. The use of partially overlapping images has been shown to improve the data quality of XCAMPO^15^. In essence, this procedure reduces the errors that arise from the imperfect Fourier sampling of individual exposures. To examine the decoding artefacts, exposure times of 15 seconds were used. At shorter exposures, the decoded images were still dominated by artefacts resulting from noise, and the weak artefacts arising from the decoding procedure were barely recognizable. The artefacts resulting from the camera noise and discrete Fourier sampling are the cause of the periodic grainy pattern observed in Fig. 4(a). This noise can be drastically reduced by using a more efficient X-ray camera (for example, a camera with fibre-optic coupling of the scintillator). The decoding artefacts are visible as weak replica or “ghost” images of the “*μ*m” symbol that are shifted relative to the real image. The extent of this shift is directly related to the period of the coding grid. A perfect reconstruction of XCAMPO images requires that the period of the coding grid *p* be larger than the focal spot size. However, this condition was not fully met in the experiment. Although the pitch of the coding grid *p* was greater than the FWHM of the focal spot, i.e., *p* > Δ*x*, the weak intensity at the tails of the focal spot led to the formation of the visible “ghost” images. Also, note that similar artefacts can be observed in Fig. 2(b), where the spurious signals are indicated by small arrows. In future experiments, this effect can be overcome by using apertures with a perfectly matched period or non-periodic masks.

### Sensitivity

**Figure 5:**
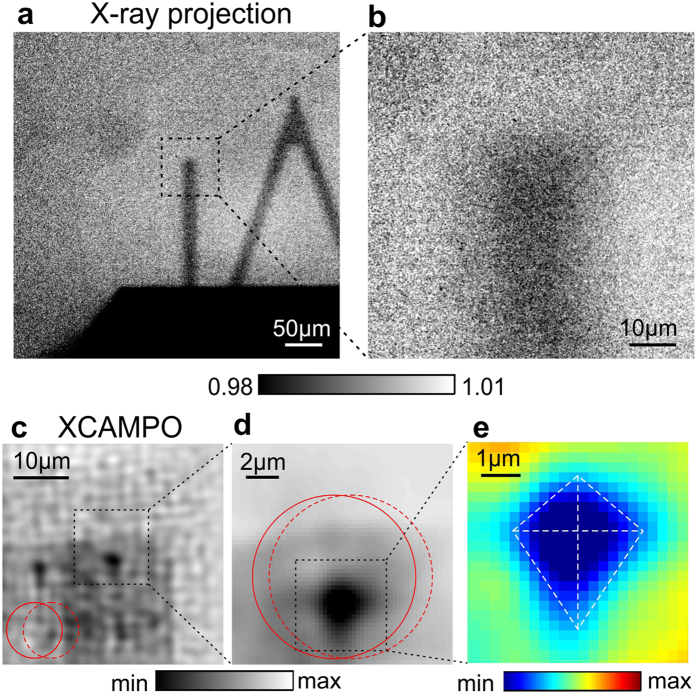
Grid-enhanced XCAMPO imaging of a weakly absorbing object (an AFM cantilever). (**a**) Standard X-ray projection image obtained with the optic but without the grid. (**b**) Zoomed-in view of (**a**). (**c**) A coarse grid-enhanced XCAMPO reconstruction performed to localize the AFM tip. (**d**) A fine grid-enhanced XCAMPO scan of the AFM tip. (**e**) Zoomed-in view of (**d**), shown using a false-colour scale for better visualization. The diamond-like shape represents a wire-frame image of the AFM tip. The centres of the red circles indicate two successive scanning positions, and their diameters reflect the focal spot size.

The process of imaging a weakly absorbing object using grid-enhanced XCAMPO was tested using an AFM cantilever (MLCT, Bruker). A conventional X-ray image of the cantilever is presented in Fig. 5(a) and (b). The cantilever was made from silicon nitride and had a nominal width of 15 *μ*m and a nominal thickness of 0.55 *μ*m. It was coated with a Ti/Au film of 45 nm in thickness. The tip had a skewed pyramidal shape with a height of 5 *μ*m. The X-ray absorption of the cantilever was less than 1%, and its width was comparable to the size of the focal spot. Hence, in the conventional X-ray projection image, the cantilever is very blurry, and the AFM tip is not visible. In the grid-enhanced XCAMPO scan, the tip can be easily localized even in the initial coarse, poor-fidelity scan shown in Fig. 5(c) (13 × 13 scan points with a 60 s acquisition time and a scan step of 3 *μ*m), which was performed to guide the movement of the tip into the focal spot. A finer scan (5 × 5 scan points with a 600 s acquisition time and a scan step of 0.8 *μ*m) clearly shows the tip. Moreover, the skewed pyramidal shape of the tip can be recognized in Fig. 5(d) and (e).

## Discussion

The novelty of the proposed grid-enhanced XCAMPO technique is that it does not rely on the specific microstructure of the polycapillary optic. Previous XCAMPO approaches have used either a periodic superstructure of capillary bundles^15^,^19^ or the intrinsic defects in polycapillary arrays^26^. Tailored polycapillaries with a defined capillary distribution are difficult to fabricate. By contrast, coding grids can be routinely prepared and optimized.

As demonstrated in Fig. 2(b), grid-enhanced XCAMPO can achieve a submicrometer half-pitch resolution. This value is lower than the resolution of the recently proposed defect-assisted imaging technique^26^, that is limited mainly by the size of a single capillary aperture. In the present experiment, the resolution was mainly limited by the detector resolution. In fact, the lens coupling of the scintillator limited the signal to approx. *f*_*c*_/4, where *f*_*c*_ is the Nyquist frequency of the detector. Hence, in principle, a resolution at the level of 0.5 *μ*m could be possible with the standard lens used in this work that was designed for micro x-ray fluorescence spectroscopy. Based on the data presented in Fig. 3 and the frequency dependence of the depth resolution in laminographic-like imaging, one can expect that for 0.5 *μ*m features, the corresponding depth resolution could be at the level of 5 *μ*m.

It is worthy to note, that in an idealized grid-enhanced XCAMPO experiment, the grid should be located exactly at the exit surface of the optics. Is such a case, the resolution is not limited by the the size of a single capillary aperture, but only by the “sharpness” of the grid or by the highest resolved spatial frequency of the grid image. This effect is somehow analogous to the lack of penumbra blur in contact imaging. However, such an idealized geometry will be hardly possible to realize in practice. Precise cutting of the optics is not straightforward and the surfaces of polycapillary devices are usually not perfectly flat. In addition, the grid thickness cannot be infinitely thin in order to provide enough stopping power of X-rays. Taking into account these limitations, one can estimate that grid-enhanced XCAMPO using a tailored optics with a shorter focal length and/or using a more efficient detector placed at a farther distance could achieve spatial resolution at the level of 250 nm–300 nm.

The obtained sub-micron spatial resolution is comparable to the resolution of indirect detection systems that use thin scintillators and which are frequently employed in synchrotron experiments^34^. However, in grid-enhanced XCAMPO, the object to be imaged is located inside the focal spot of the optics and submicron X-ray imaging could be realized simultaneously with *μ*XRF scans. Since the coding grid absorbs only approx. 50% of the primary X-ray photons, a polycapillary optics (with a grid in front of it) could be still very efficiently used for element specific *μ*XRF scans. Grid-enhanced XCAMPO could provide high-resolution transmission images of the same sample. A similar solution has been previously described as a very useful for *μ*XRF systems, that are based on monocapillary optics^27^. The main drawback of grid-enhanced XCAMPO is related to a small field-of-view, which requires scanning and recording of many overlapping images. However, scanning is inherent to *μ*XRF and the optimization of the overlap of the adjacent scan spots may shorten the data acquisition and/or improve the decoding procedure^35^. Grid-enhanced XCAMPO could be also adopted for X-ray phase-contrast imaging^36^,^37^ or dark-field imaging^38^ that is based on coded aperture masks. Also, a general concept of structural illumination^39^,^40^ could be very useful for obtaining multimodal images with polycapilary optics.

In this work, a standard TEM grid was used as a coding aperture. The use of a grid with a pitch that is better matched to the size of the focal spot will eliminate “ghost” artefacts. Due to the robustness against noise, the use of a periodic aperture and an optics with a small focus is most probably the best solution for laboratory based experiments. For a periodic mask, the deconvolution procedure is limited to intense harmonic peaks in the reciprocal space that are characterized by a high signal-to-noise ratio. In addition, the coded aperture is especially efficient when the object is small^41^ i.e. when the size of the focal spot does not exceed much the spatial resolution. However, grid-enhanced XCAMPO could be also realized with focusing polycapillary half-lenses at synchrotrons. In such a case, the signal-to-noise ratio is not a major concern and it would be interesting to perform experiments with non-periodic masks^23^,^22^,^42^. Non periodic-mask would permit a more uniform sampling of the object’s Fourier space and permit high-resolution imaging with optics that have much larger focal spots.

## Methods

### Experimental details

The experiments were performed using a tungsten anode X-ray tube (XTG5011 Apogee, Oxford Instruments) with a 40 *μ*m spot that was operated at 50 kV and 1 mA. The optics (microlens for X-ray fluorescence spectroscopy, IfG) consisted of an array of approx. 3 × 10^5^ capillaries with an intercapillary spacing of ~1.2 *μ*m^26^. It had an exit working distance *f* ≈ 2.5 mm and an exit aperture of 1.1 mm and produced a focal spot with a FWHM of Δ*x* ≈ 11 *μ*m. The total number of photons per second in the spot was ~3 × 10^8^. X-rays were recorded using a cooled sCMOS 2 k × 2 k pixel camera (Photonic Science) that was lens coupled to a Gadox (16 mg/cm^2^) scintillator with dimensions of 82 × 82 mm^2^. The effective pixel size was 42 *μ*m, but the lens-limited resolution of the system was at the level of 150 *μ*m. Note that for large scintillators, lens coupling is inefficient, and the sensitivity can be drastically improved by using fibre-optic coupling. The scintillator was placed at a distance of *D* ≈ 40 cm from the optic. The optic and the test objects were placed on piezo XYZ stages (MX35 and MS30, Mechonics). For the grid, we used a gold transmission electron microscopy 2000 mesh grid (pitch of 12.5 *μ*m, bar width of 5 *μ*m) with a thickness of 4 *μ*m. The grid was placed on a holder that could be moved by means of three stepper-motor-driven translation stages (Standa, 8MT173). The grid was placed at a distance of *g* ≈ 0.5 mm from the lens output surface (the minimum distance allowed by the presence of the beryllium window of the optic). The pinhole used in the experiments had a diameter of 1 *μ*m and was made of Mo absorbing material. Motion control, data acquisition and data analysis were performed using home-developed MATLAB procedures.

### Data analysis

The grid-enhanced XCAMPO data were decoded based on equation 1. For a single exposure, decoding was performed by means of the convolution theorem. The reconstructed image in (*ν*_*x*_, *ν*_*y*_) Fourier space was calculated as follows:


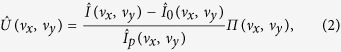


where the hat symbol denotes the Fourier transform; *I* and *I*_0_ are images of the grid recorded with and without the object present, respectively; *I*_*p*_ is an image of the grid recorded with the pinhole placed in the focal spot, which approximates the coding aperture *S*; and Π(*ν*_*x*_, *ν*_*y*_) is a mask that limits the effects of the decoding procedure to sharp peaks in Fourier space (cf. Fig. 1(e)). The real-space reconstruction *U* was obtained from the reciprocal-space image 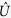 via the inverse fast Fourier transform. To increase the FOV, the sample was scanned in the lateral *xy* plane in steps of Δ, which were smaller than the lateral size of the focal spot, similar as in ptychography^43^. The final image *V* was composed of partially overlapping images *U* according to *V(x, y*) =∑_*ij*_*U*_*ij*_(*x*′, *y*′)*G(x*′, *y*′), where *x*′ = *x* − *i*Δ, *y*′ = *y* − *j*Δ, *x* and *y* are the non-magnified real-space coordinates, and *i* and *j* enumerate the scan positions. *G* is a Gaussian function with approximately the same shape as the focal spot. It was used to suppress the reconstruction of spurious signals in the region outside the focal spot.

## Additional Information

**How to cite this article:** Sowa, K. M. *et al*. Grid-enhanced X-ray coded aperture microscopy with polycapillary optics. *Sci. Rep.* **7**, 44944; doi: 10.1038/srep44944 (2017).

**Publisher's note:** Springer Nature remains neutral with regard to jurisdictional claims in published maps and institutional affiliations.
